# In multiple myeloma, monthly treatment with zoledronic acid beyond two years offers sustained protection against progressive bone disease

**DOI:** 10.1038/s41408-024-01046-2

**Published:** 2024-04-15

**Authors:** Thomas Lund, Michael Tveden Gundesen, Annette Juul Vangsted, Carsten Helleberg, Einar Haukås, Trine Silkjær, Jon Thor Asmussen, Elena Manuela Teodorescu, Bo Amdi Jensen, Tobias Schmidt Slørdahl, Hareth Nahi, Anders Waage, Niels Abildgaard, Fredrik Schjesvold, Nordic Myeloma Study Group

**Affiliations:** 1 Department of Hematology, Odense University Hospital, Odense, Denmark; 2 Department of Clinical Research, University of Southern Denmark, Odense, Denmark; 3 Department of Hematology, Rigshospitalet, Copenhagen, Denmark; 4 Department of Blood and Cancer Diseases, Stavanger University Hospital, Stavanger, Norway; 5 Department of Hematology, Aarhus University Hospital, Aarhus, Denmark; 6 Department of Radiology, Odense University Hospital, Odense, Denmark; 7 Department of Hematology, Aalborg University Hospital, Aalborg, Denmark; 8 Department of Hematology, Zealand University Hospital, Roskilde, Denmark; 9 Department of Hematology, St. Olavs Hospital and Norwegian University of Science and Technology (NTNU), Trondheim, Norway; 10 Karolinska Institutet, Stockholm, Sweden; 11 Oslo Myeloma Center, Department of Hematology, Oslo University Hospital, Oslo, Norway; 12 K.G. Jebsen Centre for B-Cell Malignancies, University of Oslo, Oslo, Norway

**Keywords:** Myeloma, Preventive medicine

Dear Editor,

Lytic bone destruction is one of the most devastating complications in Multiple Myeloma (MM). At diagnosis, more than half of patients have bone pain, and 90 percent of all patients with MM will experience bone destruction that may cause pain, pathological fractures, nerve root compression, hypercalcemia and potentially spinal cord compression [1].

Newly diagnosed symptomatic patients with MM are recommended treatment with zoledronic acid (ZOL) as part of standard anti-MM treatment [2]. ZOL has proven to reduce progression of bone disease, diminish pain, and increase survival [3]. Potential side effects to bisphosphonate treatment include kidney failure, atypical femur fractures and importantly Medication Related OsteoNecrosis of the Jaw (MRONJ) [1]. The risk of MRONJ increases with the duration of bisphosphonate treatment [4]. Early randomized studies showing effect of aminobisphosphonates administered pamidronate or ZOL monthly for up to 24 months [5, 6]. Considering the balance between benefits and risks of prolonged treatment, most guidelines recommend bisphosphonate treatment for 1–2 years as standard. With the introduction of several novel anti-MM drugs, the life expectancy for patients has increased [7]. Longer survival spurs the need to consider longer bone protective regimens. Milestone analysis after two years in the British MRC IX study showed superiority of continued ZOL treatment after 24 months. However, the comparator, clodronate given orally, was an inferior treatment, and the benefits observed after 24 months for ZOL could be caused by long-term efficacy of the treatment given for the first two years [8].

On the other hand, a study suggested that for patients with deep responses, ZOL treatment may be reduced, but the evidence is far from clear [9].

Still, both under- and overtreatment carry considerable risks. Hence, high quality evidence from randomized studies on the effect of continued treatment is important. This letter presents the main findings of the Nordic Magnolia study (EudraCT number 2014-002494-12).

To investigate the effect of ZOL in two vs. four years, we prospectively followed newly diagnosed patients with symptomatic MM. After two years of treatment, patients were randomized to either additional two years of treatment or observation. An amendment allowed patients to be included and randomized after 2 years of ZOL treatment off-protocol. Inclusion demanded symptomatic MM, estimated creatinine clearance >30 ml/min, no former bisphosphonate treatment, whereas subjects could be enrolled whether they had bone lesions or not. For full in-/ex-clusion and randomization criteria please see supplementary material. Monthly outpatient visits included: Doctors’ visits including response evaluation and fasting blood samples. ZOL was administered monthly for the duration of the study. Clinical MRONJ evaluation and Health-related Quality of Life (QoL) questionnaires (EORTC QLQ-CTC30 and QLQ-MY20) were done every three months [10]. Bone imaging was performed according to International Myeloma Working Group recommendations [11, 12] and additionally, preplanned whole body low-dose computerized tomography (WBLDCT) was performed every 6 months. WBLDCT was evaluated by local radiologists and compared to latest imaging and imaging at inclusion. Per definition, progressive bone disease (PBD) required ≥25% increase in size of osteolytic lesions or new osteolytic lesions (at least 10 mm increase/diameter), spontaneous fractures, or new compressions. For full definition, see supplementary material.

The primary endpoint was hazard rate of time to PBD calculated using Cox Regression. Secondary endpoints were time to MRONJ, overall survival (OS), progression free survival (PFS) and QoL.

At the start of the Magnolia study, there were no published data on the incidence on PBD after year 2. We assumed an annual incidence of 7.5% and a risk reduction of 50%. Therefore, 286 randomized patients were calculated to have a power of 80% at *P* < 0.05 significance level. During our trial, a parallel study with 170 patients reported an incidence of skeletal related events after 4 years of 43% vs 21% in patients treated with ZOL for 2 vs 4 years [13]. Based on these incidences, 80% power at *P* < 0.05 could be achieved with 166 randomized patients. The study review committee decided to stop further inclusion in the study, as 193 patients had been randomized.

The trial was executed in accordance with the Declaration of Helsinki and was approved by The Regional Committees on Health Research Ethics for Southern Denmark(EPN S-20140138) and Norway(EPN REK 2015/626).

## Results

**Fig. 1:**
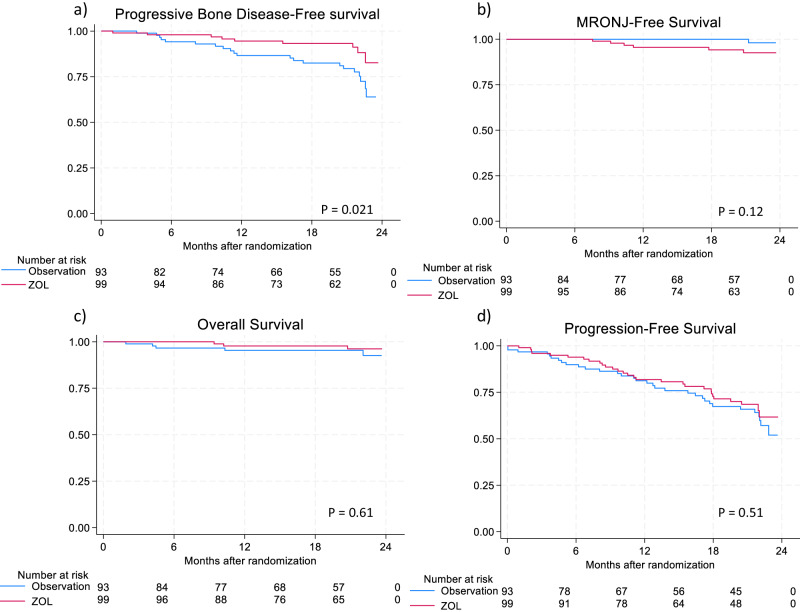
Survival curves for “time to event” for PBD, MRONJ, OS and PFS in patients with multiple myeloma randomized to either continued zoledronic acid treatment or observation after two years of monthly zoledronic acid. Milestone analysis for patients randomized 24 months after diagnosis of symptomatic myeloma. Zero months is time of randomization. **a** Progressive Bone disease (PBD), **b** Medicine related osteonecrosis of the jaw (MRONJ), **c** overall survival (OS) (**d**) progression-free survival. **a** Risk of PBD was significantly lower in the treatment group. b,c,d showed no significant difference between groups.

In total, 193 patients who had received two years of treatment with ZOL and were suitable for continued treatment (see full criteria in supplemental material), were randomized to either observation (*n* = 94) or two additional years of ZOL treatment (*n* = 99). A flowchart of inclusion and exclusion can be found in the supplementary material. The randomized groups presented with similar baseline characteristics (Table 1a). After randomization, 664 WBLDCT scans were performed. Overall, 30 cases of PBD were found after randomization (observation *n* = 21, ZOL *n* = 9). Patients treated for four years had a significantly lower risk of PBD (hazard ratio = 0.40, 95%CI (0.18%-0.87%), *p* = 0.021). The most common observed cause of PBD was “multiple new or growing lesions” (observation *n* = 16, ZOL *n* = 5; *p* = 0.007) (Table 1b). The number needed to treat to avoid one case of PBD was 7.5. Data is presented in Fig. 1a. Treatment responses to last given treatment prior to randomization in patients with PBD were: PD *n* = 1, SD *n* = 1, PR *n* = 3, VGPR *n* = 16 and CR = 7 and sCR=2.

There was no statistically significant between-group difference in the occurrence of MRONJ (ZOL: *n* = 6; Observation: *n* = 1), Fig. 1b. Importantly, only 2/6 patients experienced symptomatic MRONJ (both grade 2). There were no observed differences in PFS and OS between the two groups (Fig. 1c, d). Creatinine increase, according to CTCAE 5 criteria, was common in both groups with a tendency to be more frequent in patients in the ZOL group, but the difference was not statistically significant (ZOL 49/99 vs observation 26/94, *p* = 0.08). Two patients in the ZOL group experienced grade 3 creatinine increase, and none in the observation group. There was no difference in the observed cases of hypo- or hypercalcemia (Table 1b). The QoL questionnaires were evaluable in 142 patients. Although mean pain (0–100) was slightly higher in the observation group, 28.1 vs 23.4(NS), we did not find statistically significant differences in QoL or differences that fulfilled criteria for minimally important difference [10]. However, in patients experiencing PBD, pain increased beyond minimally important difference from 32.6/100 (baseline) to 40.5/100 (QoL questionnaire three months before) and 38.9/100 (QoL at or latest before PBD).

**Table 1:** Baseline characteristics at randomization (a) and outcomes after completed study (b) in 193 myeloma patients treated with zoledronic acid for two years and randomized to either two additional years treatment or observation.

a) Baseline characteristics	ZOL		Observation		*P* value
Number of subjects	99		94		
Age, mean (range)	67.1 years	(42–83)	65.4 years	(43–82)	0.18
Gender, male (%)	60	(60.6)	55	(59.1)	0.77
Myeloma type (%)					
IgG	58	(58.6)	58	(61.8)	0.66
IgA	19	(19.2)	18	(19.1)	0.99
Light Chain	22	(22.2)	18	(19.1)	0.28
ISS at diagnosis (%)					
I	49	(49.5)	39	(41.5)	0.26
II	31	(31.3)	38	(40.4)	0.19
III	16	(16.2)	14	(14.9)	0.81
Missing	3	(3.0)	3	(3.2)	0.95
WHO Performance status:					
0	63	(63.6)	57	(60.7)	0.89
1	29	(29.4)	30	(31.9)	0.69
2	3	(3.0)	5	(5.3)	0.43
3	2	(2.0)	2	(2.1)	0.96
Missing	2	(2.0)	0	(0.0)	0.59
Lytic bone disease at diagnosis	71	(71.7)	72	(76.5)	0.44
Autologous transplant	66	(66.7)	65	(69.1)	0.71
Treated with PI	91	(96.8)	89	(94.7)	0.44
Treatment lines (mean) (95%CI)	1,4	(1.3-1.5)	1,4	(1.3-1.5)	-
Response assesment at randomization					
sCR	17	(17.2)	14	(14.9)	0.66
CR	19	(19.2)	19	(20.2)	0.86
VGPR	41	(41.4)	40	(42.6)	0.63
PR	15	(15.2)	14	(14.9)	0.96
SD	2	(2.0)	4	(4.3)	0.37
PD	3	(3.0)	1	(1.1)	0.34
Missing	2	(2.0)	2	(2.1)	0.96
**b) Outcomes**					
Progressive Bone disease (Total)	9	(9.1)	21	(22.3)	0.021
Multiple new or growing lesions	5	(5.0)	16^a^	(17.0)	0.007
Single new or growing lesion	3	(3.0)	2	(2.1)	0.69
Non-vertebral spontaneous fracture	1	(1.0)	2^a^	(2.1)	0.53
Vertebral collapse	0	(0.0)	3^a^	(3.1)	0.11
Hypercalcemia >1.40 mmol/L^b^	0	(0.0)	0	(0.0)	1.00
MRONJ	6	(6.1)	1	(1.1)	0.12
Creatinine increase	39	(39.4)	26	(27.7)	0.08
Hypocalcemia any grade	47	(47.5)	39	(41.5)	0.40
Hypercalcemia any grade	15	(15.1)	12	(12.8)	0.63
Death	2	(2.0)	3	(3.2)	0.61

(a) Baseline characteristics of 193 patients with multiple myeloma randomized to either continued zoledronic acid treatment or observation after two years initial treatment with zoledronic acid. Patients were stratified based upon whether they had lytic bone disease at diagnosis, had received autologous transplantation and whether they had been treated with a proteasome inhibitor (PI). (b) Outcomes of randomized patients’ progressive bone disease, medication-related osteonecrosis of the jaw (MRONJ) and death were calculated using Cox Regression. The remaining outcomes were calculated using chi-squared test.

^a^2 cases of vertebral collapse and 1 spontaneous fracture was found in addition to multiple new lesions.

^b^S-Ca-ion > 1,40 mmol/L or S-Calcium adjusted for S-albumin > 2,75 mmol/L, measured in at least two consecutive blood samples.

## Discussion

This study found that continuing monthly treatment with ZOL for four years significantly decreased the risk of PBD at year three and four, with a hazard rate of 0.40.

Our finding is in accordance with the observation in the study by Avilès et al. [13]. Furthermore, the MRC Myeloma IX data showed significant reduction in skeletal related events and increased overall survival in patients who continued ZOL treatment beyond two years compared to clodronate [8]. Despite overall superiority, MRC Myeloma IX did not show superiority of ZOL compared to clodronate in regards to PBD in patients who had achieved at least CR during their latest treatment [9] Therefore, it has been suggested that ZOL treatment could be partially guided by treatment response [2]. Our data do not support this, as 9/30 patients with PBD had achieved at least CR during their last treatment. Likewise, our recent publication studying the value of preplanned WBLDCT in detecting PBD including patients from all four years of the Magnolia study demonstrated that 76% of patients with PBD had obtained VGPR or better in their latest line of treatment [14].

Prolonging treatment with ZOL monthly for four years may result in increased side effects. There was a tendency toward increased MRONJ in the treatment arm, however, there were only two cases of symptomatic (grade 2) MRONJ in total. In comparison, Avilès et. al. reported zero cases of MRONJ from year two to year four [13]. There was also a tendency towards more patients in the treatment group experiencing increased creatinine (*P* = 0.08), but only two patients left the study due to creatinine increase.

The most common observed cause of PBD was multiple new or growing lesions whereas fractures were only observed in few cases during follow-up period. However, the clinical value of prolonged bone protection is supported by the fact that patients reported increasing pain prior to PBD. Moreover, it is a fair statement that progression of lytic lesions predict and precede clinical events.

The optimal interval between ZOL infusions has not been well established. It has been suggested that ZOL every 3 months may be sufficient [2]. In a study reported in 2017, Himelstein et al. randomized patients with breast or prostate cancer with bone metastases or MM, to ZOL every 4 weeks versus every 12 weeks [15] and found no difference in skeletal events. In our data, the curves for PBD did not diverge for the first 5 months (Fig. 1a), indirectly supporting that monthly treatment may not be necessary. Limitations in our study includes slightly more osteolytic disease and ISS II in observation group, while non-significant an effect cannot be discounted. In addition, a lower sensitivity was achieved than if the planned 286 patients had been randomized.

A possible approach could be to treat patients monthly for two years and then prolong the intervals between infusions to every three month for the next two years, thereby reducing the risk of side effects and the strain on the patients. However, further studies are needed to evaluate this.

In conclusion, we found that prolonged treatment with ZOL after two years significantly reduced the risk of PBD with a hazard ratio of 0.40 with a non-significant trend towards more side effects. The main finding was fewer patients experiencing new or growing osteolytic lesions which over time may precede clinical events.

### Supplementary information

supplemental clean

Magnolia Study protocol

CONSORT Checklist

AJ-Checklist

## Data Availability

The datasets generated during and/or analyzed during the current study are not publicly available due to privacy or ethical restrictions, but are available from the corresponding author on reasonable request.
