# Supplementary material for: In multiple myeloma, monthly treatment with zoledronic acid beyond two years offers sustained protection against progressive bone disease
Source: Blood Cancer J. 2024 Apr 15;14(1):65. doi: 10.1038/s41408-024-01046-2 (PMC11018794; doi:10.1038/s41408-024-01046-2)
Supplement: Supplementary file 1 — supplemental clean [file 41408_2024_1046_MOESM1_ESM.docx]

Overview over inclusion in Magnolia protocol

Supplemental Figure 1: Overview over inclusion in Magnolia protocol.


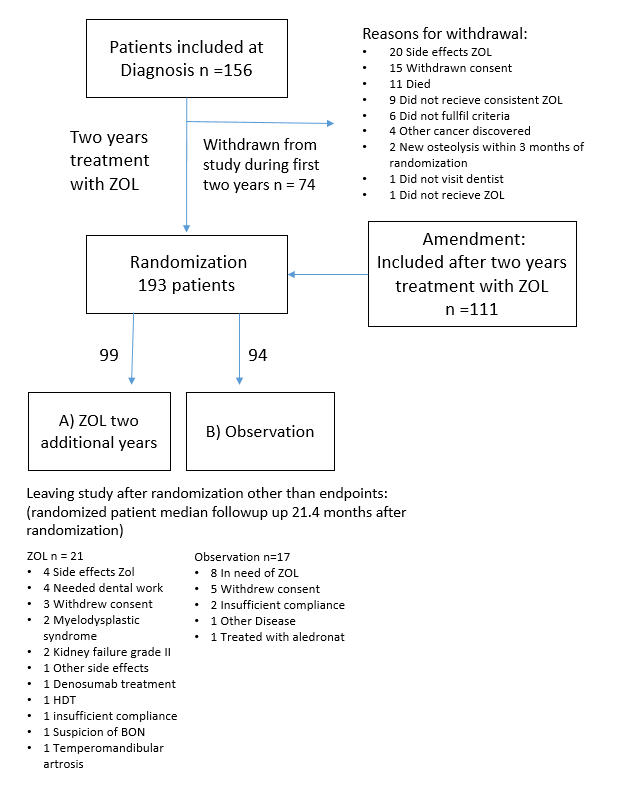


Inclusion:

Patients where included in participating centers in Denmark and Norway and study ran from from 2015-2023. Patients where stratified by having received proteasome inhibitor, whether, autologous stem cell transplant and wether they had osteolytic lesions. For each of the eight stratifications a random string of treatment/non treatment was generated. This was kept by a study nurse and each time a patient was included that study nurse would assign the next spot in the corresponding stratified group to that patient. Final endpoint was hazard ratio for PBD in treatment group and assumption of proportional hazards were tested by log-log plot.

Inclusion, exclussion and randomization in Magnolia followed the following criteria

**Inclusion criteria:**

-Symptomatic Multiple Myeloma according to the IMWG criteria, regardless of bone disease status

-Signed Informed Consent

-Age ≥ 18 years

-Remaining life expectancy ≥ 2 years

-Any concurrent anti-myeloma treatment are allowed

**Exclusion criteria of Magnolia Protocol:**

-Previous treatment with bisphosphonate within the last 6 months

-Severely reduced renal function (creatinine clearance <30 mL/min despite fluid replacement)

-Known concurrent malignancy, excluding skin cancer

-Known hypersensitivity to zoledronic acid

-Pregnant or lactating women

-Women of childbearing potential or men engaging in sexual activity with a woman of childbearing potential who refuse to use contraception (safe methods of contraception are considered to be: combined (estrogen and progestogen containing) hormonal contraception associated with inhibition of ovulation, progestogen-only hormonal contraception associated with inhibition of ovulation, intrauterine device (IUD), intrauterine hormone-releasing system ( IUS) bilateral tubal occlusion, vasectomy, and sexual abstinence. Contraception must be used until 56 days after the last infusion).

**Criteria for randomisation (at year two):**

-No severely reduced renal function (creatinine clearance <30 mL/min despite fluid replacement)

-No progressive bone disease diagnosed using bone imaging, less than or equal to three month prior to the randomization (year two)

-The patient should have received at least 12 infusions with zoledronic acid in the previous two years period. If a patient has received less than 12 infusions in the previous two years he / she it not eligible for randomisation and should leave the study.

Amendment: Patients may be included when they have received two years of zoledronic acid treatment outside clinical trials. They will proceed directly to randomization

**Inclusion criteria (amendment):**

- Earlier diagnosed with symptomatic Multiple Myeloma according to the IMWG criteria, regardless of bone disease status

-Signed Informed Consent

-Age ≥ 18 years

-Remaining life expectancy ≥ 1 years

-Received 23-25 monthly infusions with zoledronic acid

-Any concurrent anti-myeloma treatment are allowed

**Exclusion criteria (amendment):**

-Severely reduced renal function (creatinine clearance <30 mL/min despite fluid replacement)

-Known concurrent malignancy, excluding skin cancer

-Known hypersensitivity to zoledronic acid

-Pregnant or lactating women

-Women of childbearing potential or men engaging in sexual activity with a woman of childbearing potential who refuse to use contraception (safe methods of contraception are considered to be: combined (estrogen and progestogen containing) hormonal contraception associated with inhibition of ovulation, progestogen-only hormonal contraception associated with inhibition of ovulation, intrauterine device (IUD), intrauterine hormone-releasing system ( IUS) bilateral tubal occlusion, vasectomy, and sexual abstinence. Contraception must be used until 56 days after the last infusion).

- Progressive bone disease diagnosed using bone imaging, less than or equal to three month prior to the randomization

- Developed BON during earlier zoledronic acid treatment

Imaging:

Patients were examined with WBLDCT systems in use at their local hospital In Denmark or Norway. Participating centers submitted test cases. Cases were evaluated by radiologist for resolution to ensure that noise to contrast ratio was found acceptable. If shoulder motion-range allowed it participants would be placed in a supine position with arms raised above their shoulders alongside their head, The scanned field spanned from vertex/elbows to the knees. All imaging was evaluated compared to latest imaging and imaging taken at inclusion. Imaging was evaluated by a local radiologist for PBD following criteria pre-defined in the protocol.

PBD: ≥ 25% progression in size of existing osteolytic lesions (a total growth of at least 10 mm is required in the longest dimension) or vertebral fractures, new osteolytic lesions (at least 10 mm in the longest dimension) or fractures, spontaneous factures, new vertebral compression, new osteolytic lesions needing irradiation therapy or surgery, hypercalcaemia caused by the myeloma (S-Ca-ion > 1,40 mmol/L or in case S-Ca-ion is not measured: S-Calcium adjusted for S-albumin > 2,75 mmol/L), measured in at least two consecutive blood samples. PBD can be diagnosed on either conventional radiography or low-dose CT. The development however must be defined by comparing the same modality over time.
