# Supplementary material for: In multiple myeloma, monthly treatment with zoledronic acid beyond two years offers sustained protection against progressive bone disease
Source: Blood Cancer J. 2024 Apr 15;14(1):65. doi: 10.1038/s41408-024-01046-2 (PMC11018794; doi:10.1038/s41408-024-01046-2)
Supplement: Supplementary file 2 — Magnolia Study protocol [file 41408_2024_1046_MOESM2_ESM.docx]

Study Protocol

**Magnolia Study**

**Prolonged Protection from Bone Disease in Multiple Myeloma.**

**An open label phase 4 multicenter international randomised trial**

NMSG 22/14

HFE-X 14.01

Initiated on behalf of the Nordic Myeloma Study Group

EudraCT number 2014-002494-12

Table of Content.

- 1. General Information p. 3
  2. Abbreviations p. 7
  3. Background p. 8
  4. Study Rational p. 10
  5. Study Objectives p. 10
  6. Study Population p. 13
- Inclusion Criteria p. 13
- Exclusion Criteria p. 13
- Randomisation Criteria p. 13
  1. Study Design p. 13
  2. Safety Considerations and Drug Administration p. 15
  3. Side Effects p. 17

1.9.1 Definition of which AE and SAE that are to be reported p. 18

1.9.2 Definition of which SUSAR that are to be reported p. 19

- 1. Concomitant Therapy p. 20
  2. Study Drug Information p. 20
  3. Withdrawal from the Study p. 20
  4. Statistical Considerations p. 21
  5. Ethical Considerations p. 21
  6. Privacy of Personal Data and Data Handling p. 22
  7. Access to Source Data p. 23
  8. Publication Policy p. 23
  9. Data Quality Assurance p. 23
  10. Funding p. 23
  11. Timescale p. 23
  12. Insurance p. 23
  13. References p. 24

Appendix 1 Flowchart Concerning Treatment and Investigations

Appendix 2 Manual for collecting blood samples and storage

Appendix 3 Manual for conduction conventional radiography and low-dose CT

Appendix 4 Quality of Life Questionnaires A and B

Appendix 5 Summary of Product Characteristics Zoledronic Acid

Appendix 6 SOP for randomisation

**Study Protocol: Prolonged Protection from Bone Disease in Multiple Myeloma**

- 1. **General Information.**

***Project title:* Prolonged Protection from Bone Disease in Multiple Myeloma.** (Magnolia Study)

*Sponsor:*

Odense University Hospital represented by

Staff Specialist MD Ph.D. Thomas Lund, on behalf on the Nordic Myeloma Study Group

Department of Haematology

Odense University Hospital

Sdr. boulevard 29

5000 Odense C

Denmark

*Principal Investigator:*

Staff Specialist MD Ph.D. Thomas Lund

Department of Haematology

Odense University Hospital

Sdr. Boulevard 29

5000 Odense C

Denmark

*Study Secretariat:*

Hematological Research Unit

Department of Haematology

Odense University Hospital

Kløvervænget 10, 12^th^ Floor

5000 Odense C

Denmark

*National Investigator Denmark:*

Staff Specialist MD Ph.D. Thomas Lund

Department of Haematology

Odense University Hospital

Sdr. Boulevard 29

5000 Odense C

Denmark

*National Investigator Sweden:*

MD, PhD. Assis. Professor Hareth Nahi
Haematology Centre Karolinska, M54
Karolinska University Hospital, Huddinge
S-141 86 Stockholm

Sweden

*National Investigator Norway:*

Consultant in haematology MD Fredrik Schjesvold

Department of Medicine

Bærum Hospital, Vestre Viken

3004 Drammen

Norway

*Steering Committee:*

Staff Specialist MD Ph.D. Thomas Lund

Department of Haematology

Odense University Hospital

Sdr. Boulevard 29

5000 Odense C

Denmark

Associate Professor MD Hareth Nahi

MD, PhD. Assis. Professor Hareth Nahi
Haematology Centre Karolinska, M54
Karolinska University Hospital, Huddinge
S-141 86 Stockholm

Sweden

Consultant in Haematology MD Fredrik Schjesvold

Department of Medicine

Bærum Hospital, Vestre Viken

3004 Drammen

Norway

Professor MD Niels Abildgaard

Department of Haematology

Odense University Hospital

Sdr. Boulevard 29

5000 Odense C

Denmark

Senior Consultant MD, PhD Kristina Carlson

Avd 50 C, Department of Haematology

Uppsala University Hospital

S-751 85 Uppsala

Sweden

Professor MD Anders Waage

Department of Haematology

Trondheim University Hospital

Postbox 3250 Sluppen

7006 Trondheim

Norway

*Scientific advisor:*

Senior Hospital Physician Jon Thor Asmussen

Department of Radiology

Odense University Hospital

Sdr. Boulevard 29

5000 Odense C

Denmark

*Monitor:*

Denmark:

Department of Clinical Biochemistry and Pharmacology

The GCP-Unit

Odense University Hospital

J. B. Winsløws Vej 19, 2. sal

5000 Odense C

Denmark

Sweden and Norway:

FoU-centrum Skåne

Kliniskt prövningsstöd

Skånes Universitetssjukhus - Lund

221 85 Lund

Sweden

Expected initiation date 2014

Expected termination date 2020.

The study will be conducted according to the study protocol, the national legislations, and the ICH-GCP guideline.

The National Medicinal Authorities, the National Ethical Committees and the National Data Protection Agencies will be notified of the study.

The Study has been registered at clinicaltrials.gov

**1.2 Abbreviations.**

AE Adverse Event

bALP Bone specific Alkaline Phosphatase

BON Bisphosphonate Induced Osteonecrosis of the Jaw

Clcr Creatinine Clearance

CRF Case Report Form

CT Computed tomography

CTX-I C-terminal cross-linked Telopeptide of type I Collagen

ICTP C-terminal cross-linked Telopeptide of type I Collagen generated by MMPs

MGUS Monoclonal Gammopathy of Undetermined Significance

MM Multiple Myeloma

PINP Procollagen type I N-terminal propeptide

PBD Progressive Bone Disease

sPBD Symptomatic Progressive Bone Disease

SAE Severe Adverse Event

SAR Serious Adverse Reaction (expected)

SRE Skeletal Related Event

SUSAR Suspected Unexpected Serious Adverse Reactions

TRAP5b Tartrate Resistant Acid Phosphatase isotype 5b

QoL Quality of Life

**1.3 Background.**

Treatment with bisphosphonate.

Multiple myeloma (MM) is a B-cell malignancy characterized by proliferation of monoclonal plasma cells in the bone marrow. One of the most devastating complications in MM is the severe loss of bone mass observed in the majority of the patients. Bone loss can either be observed as typical osteolytic lesions or as a more generalized osteopenia. At diagnosis, 79 percent of all patients present with abnormal findings on conventional radiography either caused by osteolytic lesions, fractures, or osteoporosis; furthermore, bone pain is present in 58 percent of the patients [[1](#_ENREF_1)]. Up to 90 percent of all patients will eventually suffer from bone destruction, which may result in pain, pathological fractures, nerve root compression, or hypercalcaemia [[2](#_ENREF_2)]. Because of this, patients with MM have a severely reduced quality of life (QoL) [[3](#_ENREF_3)]. Since Berenson *et al*. in 1996 demonstrated that the use of the bisphosphonate pamidronate can reduce the number of skeletal related events (SRE) and diminish pain, bisphosphonate has been the cornerstone in treating bone disease in MM [[4](#_ENREF_4)]. In 2003, the more potent bisphosphonate zoledronic acid was demonstrated to be “non-inferior” to pamidronate in reducing SRE in MM [[5](#_ENREF_5)]. Later it was demonstrated that zoledronic acid not only reduced the number of SRE but also increased overall survival with 5.5 months [[6](#_ENREF_6)], an association that had been very difficult to demonstrate for pamidronate [[7](#_ENREF_7)]. Likewise it has been demonstrated that patients without bone disease at diagnosis; evaluated with conventional radiography; may benefit from zoledronic acid. A significant reduction in the incidence of SRE has been observed in this subgroup of patients when zoledronic acid is initiated already at diagnosis [[8](#_ENREF_8)].

Duration of bisphosphonate treatment.

In 2003 it was first suggested that treatment with bisphosphonates could cause bisphosphonate induced osteonecrosis of the jaw (BON) [[9](#_ENREF_9)]. BON is a serious disorder where the bone, usually of the mandible, starts to dissolve. The incidence in BON increases with the duration of bisphosphonate treatment and the potency of the bisphosphonate; and is commonly observed subsequently to tooth extraction [[10](#_ENREF_10), [11](#_ENREF_11)]. Because of BON, various recommendations regarding preventive initiatives have been published on how to reduce the incidence of this side effect. These preventive initiatives include; high-risk dental conditions should be treated prior to bisphosphonate initiation, good oral hygiene should be maintained throughout the treatment period, after treatment initiation invasive dental procedures should be avoided if possible, prophylactic antibiotics should be given in case of invasive dental procedures, and the duration of bisphosphonate treatment should if possible be limited [[2](#_ENREF_2), [12](#_ENREF_12)]. In combination, these preventive initiatives have reduced the incidence of BON [[13](#_ENREF_13), [14](#_ENREF_14)]. Concerning the duration of bisphosphonate treatment, most guidelines suggests two years of treatment after which further treatment should be at the discretion of the treating physician [[2](#_ENREF_2), [15](#_ENREF_15)]. The reason for the two years separation is based on very little scientific evidence. But, the two initial bisphosphonate studied had a follow-up of 21 and 24 months respectively [[5](#_ENREF_5), [7](#_ENREF_7)]. There are scarce data concerning what happens once bisphosphonate is discontinued in MM. However we do know that biochemical markers of bone resorption start to increase shortly after discontinuation, and that the rate of the increase is greater if the preceding treatment duration is short [[16](#_ENREF_16)]. Likewise there are data indicating that early re-initiation of bisphosphonate may prevent development of progressive bone disease (PBD) [[17](#_ENREF_17)]. These data could suggest that, by withholding bisphosphonate in order to avoid BON, the patient is subjected to an increased risk of bone pain, hypercalcaemia, or fractures. This schism is becoming increasingly relevant as the expected life time of the patients is increasing these years, thus resulting in a prolonged time period where the patient perhaps receives inferior treatment due to fear of potential side effects.

Bone markers in multiple myeloma.

Throughout life, bone is continuously being remodelled with removal of old bone by the osteoclast followed by replacement with new bone by the osteoblast. In MM, bone resorption is generally increased both in regard to numbers and activity of the osteoclast. Bone formation on the other hand follows a more biphasic pattern, being compensatory increased early in the disease, but later as the disease progresses it becomes depressed [[18](#_ENREF_18), [19](#_ENREF_19)]. This imbalance ultimately leads to loss of bone mass and osteolysis. Biochemical markers of bone remodelling are components that are released to the blood during the bone remodelling process. Unlike bone imaging, which provides static data, bone markers provide dynamic information on the current bone turn over rate [[20](#_ENREF_20)]. Furthermore, bone markers can be measured in a blood sample, hopefully negating the need for radiation. Bone markers can be divided into markers of bone resorption and markers of bone formation, thus evaluating each part individually. Recently we have demonstrated that changes in the levels of different bone markers preceded progressive bone disease (PBD) in the individual patient [[21](#_ENREF_21)]. We therefore hypothesis that, in the future, development in bone marker levels in the individual patient can be used to tailor bisphosphonate treatment in the individual patients, as opposed to treatment for a fixed time period in all patients. Our hope is that treatment could be initiated as soon as bone turnover becomes abnormal instead of waiting for painful osteolysis to develop. Likewise, treatment could be withheld when bone remodelling becomes stable thereby avoiding unnecessary side effects. In this study, we want to collect markers of bone resorption and bone formation and thus retrospectively be able to calculate cut of values for the different markers where the best positive- and negative predictive values are achieved. Since this parameter is partly explorative it will the needed afterwards to design prospective trials where we test the clinical usefulness of bone marker guided treatment. Another way to separate the markers is into extracellular components released during the actual bone remodelling process or into cellular components reflecting the number of osteoclasts or osteoblasts respectively. The extracellular markers profit from being highly specific but are also subject to high variation, whereas the cellular markers are less specific but more stable. We also want to use this study to investigate if it is the more specific markers of actual bone remodelling or the more stable cellular markers that are the safest to use in a future prospective clinical trial. The following bone markers will be measured: C-terminal cross-linked Telopeptide of type I Collagen (CTX-I), a bone degradation marker reflecting actual collagen breakdown. Tartrate Resistant Acid Phosphatase isotype 5b (TRAP5b), a bone degradation marker reflecting the number of osteoclasts. Procollagen type I N-terminal propeptide (PINP), a bone formation marker reflecting collagen synthesis. Bone specific Alkaline Phosphatase (bALP) a bone formation marker reflecting the number of osteoblasts, and C-terminal cross-linked Telopeptide of type I Collagen generated by MMPs (ICTP), a bone degradation marker reflecting matrixmetalloproteinase-induced collagen breakdown. ICTP will be measured only at diagnosis. For further details on when bone markers will be measured, please see section 1.7 and appendix 1.

Bone imaging in multiple myeloma.

Despite recent advances in imaging techniques, conventional radiography remains the golden standard when evaluating bone disease in MM [[22](#_ENREF_22), [23](#_ENREF_23)]. Presence or absence of osteolytic lesions may define if a patient is ultimately diagnosed with asymptomatic MM (which results in a watch and wait strategy) or active MM (which results in chemotherapeutic treatment). The rational behind this is that patients with osteolytic bone disease rapidly progress to more active disease if left untreated [[24](#_ENREF_24)]. Conventional radiography however, is known to have a low sensitivity since at least 30% of the trabecular bone substance must be lost before osteolytic lesions becomes detectable; likewise areas such as the scapula, the ribs, and the sternum can be difficult to visualise [[22](#_ENREF_22)]. Computed tomography (CT) scanning is superior to conventional radiography in detecting osteolytic lesions, however due to the high radiation exposure it seems inappropriate to use this modality as a screening tool [[25](#_ENREF_25)]. However, because of the advances in radiology it is now possible to design whole body low-dose CT protocols, reducing the radiation exposure from 25.5 mSv to 4.1 mSv (conventional radiography 2.4 mSv) without loosing the visualisation capabilities of the CT [[26](#_ENREF_26)]. Furthermore, low-dose CT is much quicker to perform and less cumbersome for the patients compared to conventional radiography of almost the entire skeleton. Few studies exists which systematically evaluate the use of low dose CT in MM. One prospective study included 39 persons diagnosed with either MM or Monoclonal Gammopathy of Undetermined Significance (MGUS) allowing both initial and restaging scannings to be included [[27](#_ENREF_27)]. A more recent study evaluated retrospectively acquired data from low-dose CT and PET-CT in 51 persons also including both initial and restaging scannings [[28](#_ENREF_28)]. Both studies found low-dose CT to be superior to conventional radiography in detecting bone disease. However, the clinical relevance of these findings remains to be determined.

**1.4 Study Rational.**

Bone disease in MM is a common disorder leading to pain, possible fractures, hypercalcaemia and a severely reduced quality of life. Treatment with zoledronic acid does not only reduce pain and the risk of SRE but also increase overall survival. Prolonged use however, increases the risk of BON, especially in patients with reduced dental health who undertakes invasive dental procedures. No data are currently available concerning the optimal duration of zoledronic acid treatment, but two years is the most common recommendation since the original studies had a follow-up of approximately 24 months. There are data emerging that indicate that cancellation of treatment results in an increased risk of PBD.

Another completely different alternative when evaluating bone disease in MM is to use biochemical markers. This method is completely without radiation exposure, and it may in the future decide when zoledronic acid should be given and when it should be withheld, thereby reducing the risk of BON without increasing the risk of PBD.

The golden standard when evaluating bone disease in MM is still conventional radiography. Never the less, conventional radiography is a cumbersome and time-consuming investigation for the patient who usually suffers from bone pain. Furthermore, lesions, especially in the axial skeleton, can be difficult to visualise and it has been demonstrated that CT is a more sensitive modality. It is now possible to conduct low-dose CT reducing the radiation exposure to an acceptable level; indicating that it should be the modality of choice. Prospective studies however are still lacking before the precise role of low-dose CT in MM can be defined.

The purpose of this study is threefold. To determine the optimal duration of treatment with zoledronic acid. To investigate if the future role of serum bone markers in MM could be to tailor treatment to the individual patient. Finally, to evaluate if low-dose CT should replace conventional radiography as the golden standard when evaluating bone disease in MM.

Amendment: After the Nordic Myeloma Study Group began working on this protocol, zoledronic acid is becoming the preferred bisphosphonate outside clinical trials in the Nordic Region. As a consequence there are now patients outside clinical trials who have received two years of zoledronic acid treatment. These patients may be included in the trial when they have received 24 infusion with zoledronic acid. They will be randomised at the time of inclusion.

**1.5 Study Objectives.**

Endpoint definitions.

PBD:

≥ 25% progression in size of existing osteolytic lesions (a total growth of at least 10 mm is required in the longest dimension) or vertebral fractures, new osteolytic lesions (at least 10 mm in the longest dimension) or fractures, spontaneous factures, new vertebral compression, new osteolytic lesions needing irradiation therapy or surgery, hypercalcaemia caused by the myeloma (S-Ca-ion > 1,40 mmol/L or in case S-Ca-ion is not measured: S-Calcium adjusted for S-albumin > 2,75 mmol/L), measured in at least two consecutive blood samples.

PBD can be diagnosed on either conventional radiography or low-dose CT. The development however must be defined by comparing the same modality over time. The modality used at the time of randomisation (year 2) will be used as baseline value.

sPBD:

Spontaneous factures, new vertebral compression, new osteolytic lesions needing irradiation therapy or surgery, hypercalcaemia caused by the myeloma (S-Ca-ion > 1,40 mmol/L or in case S-Ca-ion is not measured S-Calcium adjusted for S-albumin > 2,75 mmol/L), measured in at least two consecutive blood samples).

sPBD can be diagnosed on either conventional radiography or low-dose CT. The development however must be defined by comparing the same modality over time. The modality used at the time of randomisation (year 2) will be used as baseline value.

Bone healing:

≥ 25% reduction in the size of osteolytic lesions (a reduction of at least 10 mm in the longest dimension is required), increased sclerosis in the edge of existing lesions, or healing of existing lesions. Bone healing can be diagnosed on either conventional radiography or low-dose CT. The development however must be defined by comparing the same modality over time.

The modality used at the time of randomisation (year 2) will be used as baseline value.

BON:

To diagnose BON the following criteria must be fulfilled

1: Current or previous treatment with a bisphosphonate AND

2: Exposed, necrotic bone in the maxillofacial region that has persisted for more that 8 weeks AND

3: No history of radiation therapy to the jaws [[29](#_ENREF_29)]

No bone disease / Bone disease:

Any radiological signs of bone disease including osteolysis (at least 10 mm), osteopenia, halisteresis, fractures including vertebral compression fractures (these findings are not necessarily equal to a Myeloma Defining Bone Disease Event; which is defined according to the International Myeloma Working Group Consensus Statement Paris 2011). No bone disease / Bone disease can be diagnosed on either conventional radiography or low-dose CT

QoL:

Quality of Life is evaluated using the European Organization for Research and Treatment of Cancer Quality of Life Questionnaire-Core 30 (EORTC QLQ-C30, version 3.0, together with the MY20 multiple myeloma specific supplement.

Primary Endpoint:

To compare the time to PBD from year 2 to year 4 in patients treated with monthly zoledronic acid in two consecutive years compared to patients treated with monthly zoledronic acid in four consecutive years.

Secondary Endpoints:

To compare the incidence of PBD from year 2 to year 4 in patients treated with monthly zoledronic acid in two consecutive years compared to patients treated with monthly zoledronic acid in four consecutive years.

To compare the incidence of sPBD from year 2 to year 4 in patients treated with monthly zoledronic acid in two consecutive years compared to patients treated with monthly zoledronic acid in four consecutive years.

To compare the incidence of bone healing from year 2 to year 4 in patients treated with monthly zoledronic acid in two consecutive years compared to patients treated with monthly zoledronic acid in four consecutive years.

To compare the overall survival from year 2 to year 4 in patients treated with monthly zoledronic acid in two consecutive years compared to patients treated with monthly zoledronic acid in four consecutive years.

To compare the incidence of BON from year 2 to year 4 in patients treated with monthly zoledronic acid in two consecutive years compared to patients treated with monthly zoledronic acid in four consecutive years.

To compare the development in QoL from year 2 to year 4 in patients treated with monthly zoledronic acid in two consecutive years compared to patients treated with monthly zoledronic acid in four consecutive years.

To compare the development in the bone markers CTX-I, PINP, bALP, and TRAP5b from year 2 to year 4 in the patients groups randomised to receive two or four years of treatment with zoledronic acid

To investigate if monthly measurements of the bone markers CTX-I, PINP, bALP, TRAP5b, or the ratios of these can be used to predict the development of PBD from year 2 to year 4.

To investigate the consistency between the finding “no bone disease” when using conventional radiography compared to low-dose CT at diagnosis*.

To investigate if the patients with inconsistency in bone disease status using the different imaging modalities at diagnosis are more likely to progress in bone disease compared to patients diagnosed with “no bone disease” using both modalities*.

To investigate if the three patients groups 1. consistent “no bone disease” 2. inconsistent bone disease 3. consistent “bone disease”, at diagnosis can be identified using the levels of the bone markers CTX-I, PINP, bALP, TRAP5b, ICTP, or the ratio of these.

* Sites may participate in the study without conduction both conventional radiography and low-dose CT.

Amendment: The primary endpoint will not be affected by the amendment. The eight first secondary endpoints will not be affected by the amendment. The last three secondary endpoints will be affected by the amendment, but it is without statistical relevance; see section 1.13

**1.6 Study Population.**

The study population comprises 358 patients with newly diagnosed symptomatic multiple myeloma. Patients with myeloma are in the Nordic countries treated in haematological clinics The patients will be recruited from participating haematological clinics within the NMSG. All patients will be invited to participate in the protocol if they fulfil the inclusion- and exclusion criteria´s. The patient will be informed of the protocol by the treating physician orally and receive written information in their own language concerning the protocol, according to the ICH-GCP guidelines. If possible, the patient will be encouraged to bring a family member to the information meeting. If needed, the patient will receive time for considerations before deciding if he/she wishes to participate in the protocol. If the patient decides not to participate in the protocol he/she will receive the clinics standard treatment.

Inclusion criteria:

-Symptomatic Multiple Myeloma according to the IMWG criteria, regardless of bone disease status

-Signed Informed Consent

-Age ≥ 18 years

-Remaining life expectancy ≥ 2 years

-Any concurrent anti-myeloma treatment are allowed

Exclusion criteria:

-Previous treatment with bisphosphonate within the last 6 months

-Severely reduced renal function (creatinine clearance <30 mL/min despite fluid replacement)

-Known concurrent malignancy, excluding skin cancer

-Known hypersensitivity to zoledronic acid

-Pregnant or lactating women

-Women of childbearing potential or men engaging in sexual activity with a woman of childbearing potential who refuse to use contraception (safe methods of contraception are considered to be: combined (estrogen and progestogen containing) hormonal contraception associated with inhibition of ovulation, progestogen-only hormonal contraception associated with inhibition of ovulation, intrauterine device (IUD), intrauterine hormone-releasing system ( IUS) bilateral tubal occlusion, vasectomy, and sexual abstinence. Contraception must be used until 56 days after the last infusion).

Criteria for randomisation (at year two):

-No severely reduced renal function (creatinine clearance <30 mL/min despite fluid replacement)

-No progressive bone disease diagnosed using bone imaging, less than or equal to three month prior to the randomization (year two)

-The patient should have received at least 12 infusions with zoledronic acid in the previous two years period. If a patient has received less than 12 infusions in the previous two years he / she it not eligible for randomisation and should leave the study.

Patients who fail to be included in the study will be treated according to the local standards. Patients who will be excluded from the study because they fail to meet the criteria for randomisation (at year two) will likewise be treated according to the local standards. Radiological data from these patients will still be used to evaluate secondary endpoints in the study. No additional protocol related follow-up is planned once the patients leaves the protocol either timely or untimely. Future treatment will be decided by the local hospital and the patient.

Amendment: Patients may be included when they have received two years of zoledronic acid treatment outside clinical trials. They will proceed directly to randomisation

Inclusion criteria (amendment)

- Earlier diagnosed with symptomatic Multiple Myeloma according to the IMWG criteria, regardless of bone disease status

-Signed Informed Consent

-Age ≥ 18 years

-Remaining life expectancy ≥ 1 years

-Received 23-25 monthly infusions with zoledronic acid

-Any concurrent anti-myeloma treatment are allowed

Exclusion criteria (amendment) :

-Severely reduced renal function (creatinine clearance <30 mL/min despite fluid replacement)

-Known concurrent malignancy, excluding skin cancer

-Known hypersensitivity to zoledronic acid

-Pregnant or lactating women

-Women of childbearing potential or men engaging in sexual activity with a woman of childbearing potential who refuse to use contraception (safe methods of contraception are considered to be: combined (estrogen and progestogen containing) hormonal contraception associated with inhibition of ovulation, progestogen-only hormonal contraception associated with inhibition of ovulation, intrauterine device (IUD), intrauterine hormone-releasing system ( IUS) bilateral tubal occlusion, vasectomy, and sexual abstinence. Contraception must be used until 56 days after the last infusion).

- Progressive bone disease diagnosed using bone imaging, less than or equal to three month prior to the randomization

- Developed BON during earlier zoledronic acid treatment

**1.7 Study Design.**

All patients included in the study will receive zoledronic acid every 4^th^ week for two years. Patients who after two years of treatment meet the eligibility criteria will be randomised (1:1) to stop treatment with zoledronic acid and enter the observation arm (treatment arm A) or to receive zoledronic every 4^th^ week up to a total period of four years (treatment arm B). Randomisations will be conducted when the patient has been in the study for two years (+/- 1 month). The randomisation procedure will be stratified according to previous autologous stem cell treatment, presence or absence of bone disease at diagnosis, previous treatment with bortezomib.

Treatment with zoledronic acid can be started before initial bone imaging has been conducted. Blood samples for measurement of fasting serum bone markers however must be collected prior to zoledronic acid treatment and anti-myeloma treatment.

Acquiring source data and planned visits.

Inclusion:

The patient must meet the specified inclusion and exclusion criteria’s.

Bone imaging must not be done more that two months prior to inclusion and should be done no more than one month after inclusion.

Fasting serum bone markers must be collected prior to treatment with zoledronic acid as well as prior to treatment with anti-myeloma treatment. Serum bone markers do not need to be collected prior to bone imaging. In case of a more than two weeks delay between initial bone marker measurements and actual infusion of zoledronic acid, new serum markers should be collected prior to the first infusion.

QoL questionnaire QLQ-C30 and MY20 should be completed no more that 2 weeks after inclusion.

Infusion with zoledronic acid should be initiated as soon as possible. However, allowing time to dental examination and possible tooth extraction and healing, zoledronic acid infusions should not be initiated later than three months after initiation of anti-myeloma treatment. Dental examination will be conducted according to local guidelines.

Planned visits year 0-2:

Bone imaging will be conducted after one year and after two years of treatment. Both time-points can be moved +/- 1 month. Bone imaging should be conducted using both conventional radiography and low-dose CT. Sites however may participate in the study without conducting both conventional radiography and low-dose CT. In that case, the chosen modality must be used consistently throughout the entire study period.

Fasting serum bone markers will be collected bi–annually. All time-points can be moved +/- 2 weeks.

Measurements of creatinine and calcium may be conducted up till 2 weeks before the visit

QoL questionnaire QLQ-C30 and MY20 will be used bi-annularly. All time-points can be moved +/- 2 weeks.

Infusion with zoledronic acid should be given ever 4^th^ week (+/- 1 week). Infusion can be delayed for up to 2 months e.g. due to temporarily impaired renal function (meaning a three month pause between two infusion is allowed). If infusions cannot be given within this time period the patient will be excluded from the study. If infusions are delayed for a significant period, it is the time passed and not the number of infusions that is relevant as to when stratification occurs.

Randomisation:

At year 2 patients will be randomised to A) stop treatment with zoledronic acid or to B) continue treatment with monthly infusions of zoledronic acid for another 2 years period (4 years in total). Randomisation is allowed to be moved -1months/+2months. The randomisation procedure will be stratified according to previous autologous stem cell treatment, presence or absence of bone disease at diagnosis, and previous treatment with bortezomib. Randomisation will take place at The Hematological Research Unit, Odense University Hospital, Denmark. For further details see appendix 6 (SOP for randomisation).

Planned visits, year 2-4:

Bone imaging will be conducted after 2½ years, after 3 years, after 3½ years, and after 4 years. All time-points can be moved +/- 1 month. In case of myeloma relapse requiring active treatment, new bone imaging should be conducted within a one-month time frame of initiation of new anti-myeloma treatment. If bone imaging has been conducted as part of relapse evaluation no less than three month prior to routine bone imaging time point,no new bone imaging need to be conducted. If any bone imaging, conducted from year 2 to 4, reveals PBD in patients not receiving zoledronic acid and the treating physician find it necessary to reinitiate treatment with zoledronic acid, the patient will be excluded from the study. In that case, fasting serum bone markers should be measured prior to re-initiation of zoledronic acid treatment. If bone imaging, conducted as part of a myeloma relapse evaluation in patients not receiving zoledronic acid, results in “no PBD”, treatment with zoledronic acid should *not* be re-initiated.

Bone imaging should be conducted using both conventional radiography and low-dose CT. Sites however may participate in the study without conducting both conventional radiography and low-dose CT. In this case, the chosen modality must be used consistently throughout the entire study period.

Fasting serum bone markers should be collected every fourth week (+/- 1 week) throughout this period. Serum samples do not need to be collected at the exact date of zoledronic acid infusion but can be collected up to two weeks prior to infusion; serum samples however must not be collected after zoledronic acid infusion. Measurements of creatinine and calcium may be conducted up till 2 weeks before the visit

QoL questionnaire QLQ-C30 and MY20 will be used every 3^rd^ month: after 27, 30, 33, 36, 39, 42, 45, and after 48 months. All time-points can be moved -2 weeks.

Infusion with zoledronic acid in the treatment arm should be given ever 4^th^ week (+/- 1 week). Infusion can be delayed for up to 2 months e.g. due to temporarily impaired renal function (meaning a three month pause between two infusion is allowed). If infusions cannot be given within this time period the patient will be excluded from the study.

For specific instructions concerning conventional radiography and low-dose CT, see appendix 2

For specific instructions concerning serum bone makers, see appendix 3

Amendment:

Patients who are included after 23-25 zoledronic acid infusions outside protocol will move directly to randomisation. The randomisation procedure will be stratified according to previous autologous stem cell treatment, presence or absence of bone disease at diagnosis, and previous treatment with bortezomib. Randomisation will take place at The Hematological Research Unit, Odense University Hospital, Denmark. For further details see appendix 6 (SOP for randomisation).

Acquiring source data (amendment):

Bone imaging must not be done more than two months prior to inclusion and should be done no more than one month after inclusion.

Fasting serum bone markers must be collected no more that 2 weeks after inclusion.

QoL questionnaire QLQ-C30 and MY20 should be completed no more that 2 weeks after inclusion.

Investigation according to local guideline for any pre-existing BON must be conducted before inclusion in the protocol amendment

Planned visits, year 2-4: (amendment)

No changes compared to original description.

**1.8 Safety Considerations and Drug administration.**

The most clinically relevant side effects to zoledronic acid include impaired renal function, hypocalcaemia, and BON. Because of this, evaluation of renal function and supplementary intake of calcium +/- vitamin D should be conducted in accordance with local guidelines. During the study, serum creatinine and ionized calcium will be measured monthly and recorded in the CRF.

Risk of BON

Concerning BON, patients should receive a thorough dental examination prior to treatment initiation if possible, maximal dental hygiene should be maintained and conservative approaches should be applied if dental problem arises. Oral investigation will be conducted at least once a year in the first two years and every 3 months from year 2 to 4 by the treating physician in order to detect any cases of BON. All cases of BON will be recorded in the CRF. All cases of BON will be reported annually to the sponsor. In case of BON, it is up to the treating physician to decide if it is in the best interest of the patient to remain on bisphosphonate treatment or if treatment should be stopped, in which case the patient is excluded from the study.

In case dental problems arise, they shall if possible be treated conservatively. No data exists as to whether zoledronic acid should be withheld or continued if tooth extraction cannot be avoided. If the treating clinician finds in the best interest of the patient, infusion with zoledronic acid may be withheld up to one month prior to tooth extraction, and up to maximum three months after tooth extraction, thus allowing a four-month pause in total from zoledronic acid treatment. It is recommended however that the patients receive prophylactic antibiotic in case tooth extraction cannot be avoided [[12](#_ENREF_12)].

Risk of PBD

Zoledronic acid is administrated to the patients in order to minimise the risk of PBD. In this study, we want to evaluate if the potential benefit of prolonged zoledronic acid treatment outweigh the increased risk of BON. To minimise the risk of undetected PBD we will from year 2 to 4 conduct regular bone imaging every 3 month. Furthermore, it is mandatory to conduct bone imaging in case of progressive myeloma disease requiring treatment. To further improve the safety of the patients we have invented a less rigours event definition (PDB) compared to the more strict definition (sPBD) where the progression have to be symptomatic to be calculated as an event. All cases of sPBD will be reported annually to the sponsor.

Radiation exposure:

The following text applies to patients in which evaluation of bone disease is conducted with both conventional radiography and low-dose CT, thus it is the patients who receive the highest radiation dose.

Scheduled bone imaging will be conducted seven times during the four years period. Perhaps one extra bone imaging will be conducted in case of relapse from year 2-4. This equals a total radiation dose of 45.5-52 mSv. A patient not included in the study would probably within the same time frame have approximately three conventional radiography and 1-2 low dose CT conducted, equal to a total radiation dose of 11.3-15.4mSv. The patients will over a four years period receive an excess radiation dose of 33.2-36.6, which is equivalent to the dose received from 1.5 conventional CT. The additional bone imaging will not only be conducted for scientific reasons, but also to make sure no serious progressive bone disease occurs when the patient is not receiving zoledronic acid.

Drug administration and renal function

Four mg of zoledronic acid will be administered intravenously at the hospital every fourth week (+/- 1 week) according to local instructions, for either two or four years.

If no local instruction exists, please see the Summary of Product Characteristics at EMEA. In brief it states: *zoledronic acid 4 mg should be given as a single intravenous infusion in no less than 15 minutes. To prevent hypocalcaemia patients should also be administered an oral calcium supplement of 500 mg and 400 IU vitamin D daily. When initiating treatment with zoledronic, serum creatinine and creatinine clearance (CLcr) should be determined. CLcr is calculated from serum creatinine using the Cockcroft-Gault formula. Zoledronic is not recommended for patients presenting with severe renal impairment prior to initiation of therapy, which is defined for this population as CLcr < 30 ml/min. In patients presenting with mild to moderate renal impairment defined as CLcr 30–60 ml/min, the following zoledronic acid dose is recommended:*

| **Baseline creatinine clearance (ml/min)** | **Zoledronic acid recommended dose*** |
| --- | --- |
| > 60 | 4.0 mg zoledronic acid |
| 50–60 | 3.5 mg* zoledronic acid |
| 40–49 | 3.3 mg* zoledronic acid |
| 30–39 | 3.0 mg* zoledronic acid |

- Doses have been calculated assuming target AUC of 0.66 (mg•hr/l) (CLcr = 75 ml/min). The reduced doses for patients with renal impairment are expected to achieve the same AUC as that seen in patients with creatinine clearance of 75 ml/min.

*Following initiation of therapy, serum creatinine should be measured prior to each dose of zoledronic acid and treatment should be withheld if renal function has deteriorated. In the clinical trials, renal deterioration was defined as follows:*

*- For patients with normal baseline serum creatinine (< 1.4 mg/dl or < 124 μmol/l), an increase of 0.5 mg/dl or 44 μmol/l;*

*- For patients with abnormal baseline creatinine (> 1.4 mg/dl or > 124 μmol/l), an increase of 1.0 mg/dl or 88 μmol/l.*

*In the clinical studies, zoledronic acid treatment was resumed only when the creatinine level returned to within 10% of the baseline value. Zoledronic acid treatment should be resumed at the same dose as that given prior to treatment interruption.*

For further details see appendix 5.

If infusion of bisphosphonate for any reason is delayed for more that two months (meaning more that three months between two infusions) e.g. due to deteriorating renal function the patient will be excluded from the study.

Amendment:

Patients included according to the amendment will have a smaller radiation exposure 32.5-39 mSv instead of 45.5-52 mSv

**1.9 Side Effects.**

Zoledronic acid has been approved by EMEA since 2001 for prevention of SRE in adult patients with advanced malignancies involving the bone. It has been used extensively for many years and has a well-known toxicity profile. It is not financially possible to conduct a new study where all possible side effects will be reported rigorous, as no pharmaceutical companies have shown interest in the study. Furthermore, it is unlikely that new – still unknown – side effects can be identified from this study since all patients most likely will receive multiple lines of anti-myeloma chemotherapeutics that will differ from site to site, and a significant portion of the patients will die due to progressive disease during the protocol. However, the Nordic Myeloma Study Group finds it very relevant to conduct a study with focus on BON, which is a late occurring, severely disabling side effect where the incidence increases proportionally to the exposure time. In this study, we will focus on BON and renal insufficiency as this is a common clinically relevant side effect to zoledronic acid. Patients will furthermore be evaluated using QoL, which ultimately will answer the question whether prolonged treatment with zoledronic acid is of benefit for these severely ill patients. All AEs and SAEs will be documented in the patient’s medical journal but will not be reported in the CRF if they can be related to the myeloma disease or to the chemotherapeutic treatment the patient receives. All SAR and SUSAR will be reported in the CRF. The treating physician decides if an AE or SAE is related to the investigational compound and thus should be reported as an SAR / SUSAR. Summary of Product Characteristics supplied in appendix 5 will be used as reference document to decide if a SAR should be upgraded to a SUSAR. All cases of renal insufficiency, BON, hypo-, and hypercalcaemia, will be reported in the CRF regardless of causality.

Registration of predefined (section 1.9.1 and 1.9.2) AEs, SAEs, SAR, and SUSARs will continue until the last infusions at year 4 +30 days for the treatment group or until year 4 +30 days for the observation group.

All national SAE, SAR, and SUSARs reported in the CRF during the trial will be listed and reported yearly by the national investigator to the National Health Authority and to the National Ethics Committee according to the national regulations by the national study secretary.

All SUSARs will be listed and reported yearly by the sponsor to the national investigators and to the Danish Health Authorities.

Annually the steering committee will discuss the numbers of SAE, SAR, and SUSARs in the two patient arms. Based on these data the committee will decide if the study should be terminated before time. Likewise an interim analysis will be conducted when half the patients have completed the study. If significant relevant different is observed already at this time-point the study will also be terminated for the benefit of the remaining patients

**1.9.1 Definitions of which AE and SAE that are to be reported in the CRF**

In this study, all cases of BON, renal insufficiency, hypo-, and hypercalcaemia will be reported as an Adverse Event (AE) or a Serious Adverse Event (SAE). An adverse event is any new, undesirable medical occurrence or change of an existing condition, whether or not it is considered related to the treatment. A serious adverse event includes, but may not be limited to:

-Death due to study medication

-Life threatening complications

-Inpatient hospitalisation or prolongation of existing hospitalisation

-Significant and persistent disability / incapacity

-An event that may require medical or surgical intervention to prevent one of the outcomes list above

In this study, temporary renal insufficiency that can be reversed either by fluid replacement or by treatment reduction will be perceived as an AE, persistent renal insufficiency will be perceived as a SAE. Persistent grade 2 BON or any grade 3 BON will be perceived as a SAE

Renal insufficiencies, hypo-, and hypercalcaemia:

Renal insufficiency hypo-, and hypercalcaemia AEs will be graded according to the National Cancer Institute Common Toxicity Criteria for Adverse Evens (NCI CTCEA) version 4.0. All AEs should be reported and related to the study drug administration as follows:

Not related:

An adverse event, which is not related to the use of the drug.

Unlikely/doubtful:

An adverse event for which an alternative explanation is more likely, e.g., concomitant drug(s),

concomitant disease(s), or the relationship in time suggests that a causal relationship is unlikely.

Possible:

An adverse event, which might be due to the use of the drug. An alternative explanation, e.g., concomitant drug(s), concomitant disease(s), is inconclusive. The relationship in time is reasonable,therefore, the causal relationship cannot be excluded.

Probable:

An adverse event, which might be due to the use of the drug. The relationship in time is suggestive (e.g., confirmed by repetition). An alternative explanation is less likely, e.g., concomitant drug(s), concomitant disease(s).

Definitive/very likely:

An adverse event that is listed as a possible adverse reaction and cannot be reasonably explained by

an alternative explanation, e.g., concomitant drug(s), concomitant disease(s). The relationship in

time is very suggestive (e.g., it is confirmed by de-challenge and re-challenge).

BON:

BON is a rare occurring event and should always be reported as at least possible related. If the patient has received radiation therapy to the neck or mandibular region one should keep the differential diagnosis of osteoradionecrosis in mind. If the treating clinician discovers cases of osteoradionecrosis this should still be reported as a possible case of BON.

Bon will be graded according to the 2009 AAOMS position paper on BON [[30](#_ENREF_30)].

Stage 0: No clinical evidence of necrotic bone, but nonspecific clinical findings and symptoms.

Stage 1: Exposed and necrotic bone in asymptomatic patients without evidence of infections.

Stage 2: Exposed and necrotic bone associated with infections as evidenced by pain and erythema in region of exposed bone with or without purulent drainage.

Stage 3: Exposed and necrotic bone in patients with pain, infection, and one or more of the following: exposed and necrotic bone extending beyond the region of alveolar bone, (ie, inferior border and ramus in the mandible, maxillary sinus and zygoma in the maxilla) resulting in pathologic fracture, extraoral fistula, oral antral/oral nasal communication, or osteolysis extending to the inferior border of the mandible or the sinus floor.

Exceptions to AE and SAE reporting.

Hospitalisations or prolongation of hospitalisation due to progression of MM or well-known complications to multiple myeloma e.g. infection or anaemia do not need to be reported to the study secretariat, nor must a SAE report be completed. It also applies to complications to administration of myeloma treatment, other elective measures or for social reasons. Death due to progression of multiple myeloma do not need to be reported as an SAE.

All SAR (1.9) and all selected SAE (1.9.1) must be reported to the study secretary within one month of discovery or notification of the event. The following data must be assigned: description, dates of onset and resolution, severity, assessment of relatedness to study treatment, other suspected drugs or devices, and action taken. The investigator may be asked to provide follow-up information. It will be left to the investigator’s clinical judgement whether or not an adverse event is of sufficient severity to require that the patient should be removed from treatment. A patient may also voluntarily withdraw from treatment due to what he or she perceives as an intolerable adverse event. If either of these occurs, the patient should undergo an end of study assessment, in which reason for end of study should be noted and be given appropriate care under medical supervision until symptoms cease or the condition becomes stable.

**1.9.2 Definitions of which SUSAR that are to be reported**

All Suspected Unexpected Serious Adverse Reaction (SUSAR) possibly related to the study drug shall be reported to the sponsor. A SUSAR is a suspected unexpected Adverse Reaction which occur in the trial and that are both unexpected and serious. Suspected expected adverse reactions (SAR) are those AEs of which a reasonable causal relationship are known and described in medicinal product, of the compounds the patients is currently receiving, are not to be reported.

All SUSARs must be reported to the national study secretary within 24 hours of discovery or notification of the event. The following data must be assigned: description, dates of onset and resolution, severity, assessment of relatedness to study treatment, other suspected drugs or devices, and action taken. The investigator may be asked to provide follow-up information.

A SUSAR resulting in death or judged as life threatening must be reported to the sponsor, the other national investigators, the regulatory authorities and the ethical committee within 7 days after the national investigator has been notified about the event. A full report has to be sent to the national authorities within 15 days by the national study secretary.

A SUSAR, which is not resulting in death or is life threatening, has to be reported to the sponsor, the other national investigators, the regulatory authorities and the ethical committee within 15 days after the national investigator has been notified about the event. A full report has to be sent by the national study secretary to the national authorities as soon as possible.

**1.10 Concomitant Therapy.**

All concomitant therapy are permitted, however caution is advised when zoledronic acid is given together with aminoglycosides, non-steroid anti-inflammatory drugs (NSAID), or other potential nephrotoxic drugs, as both MM in itself and zoledronic acid are potentially nephrotoxic.

**1.11** **Study Drug Information.**

The study drug, zoledronic acid, is commercially available and approved by EMEA. The study drug is given as intravenous infusion at the local hematologic department and handled according to local routine. No study drugs are handed over to the patients. For details, see appendix 5

**1.12 Withdrawal from the Study.**

In accordance with the current revision of the Declaration of Helsinki and applicable regulations, a

patient has the right to withdraw from the study at any time for any reason without prejudice to

his/her future medical care by the physician or at the institution.

Patients may be excluded from study in case of:

-significant protocol violation or non-compliance

-refusal of the patient to continue treatment and observations; withdrawal of consent

-if the investigator believes that for safety reasons (e.g., adverse events), it is in the best interest of the subject to stop treatment.

-if the patient due to renal failure is unable to receive zoledronic acid in two consecutive months (infusion can be delayed for up to three months). In case of tooth extraction, a delay of up to four months is allowed.

-if the patient due to BON stops treatment with zoledronic acid.

-if patients in the control arm experience progressive bone disease and the treating physician find it in the best interest of the patient to reinitiate treatment with zoledronic acid.

-if the subject becomes pregnant

-death

Patients excluded from the study will be followed for 30 days by the local hospital to insure registration of late occurring AE, SAE, SAR and SUSARs

The date and the reason for withdrawal status should be stated in the case report form. If the patient withdraws his or / her consent no further investigations will be conducted. If the patient is excluded for other reasons and it poses no risk for the patient fasting serum bone marker should be measured upon withdrawal, and bone imaging should be conducted no less than one month after withdrawal from the study. Data collected from the patient up until the time of withdrawal will if possible be used in the final evaluation of the study result unless the patient states it otherwise.

As stated in section 1.13, 286 patients need to be randomised to achieve enough power in the study. Patients will be included until this number is reached

**1.13 Statistical Considerations.**

Primary endpoint:

There are no published data on the incidence on PBD from year 2 and onward in MM. However, there are data showing the annual incidence of the more strict sPBD is approximately 5% in patients receiving continuous zoledronic acid [[31](#_ENREF_31)]. Since we in this study use a less strict definition of progression in bone disease, we assume the annual incidence will be 7.5% instead of only 5% in patients receiving zoledronic acid. Based on data from the MRC IX trial and from an ASH publication in 2012 we expect to see risk reduction rate of 0.5 [[32](#_ENREF_32)]. With a significance threshold of 0.05, one-sided, a power of 80, and a fixed study duration of 2 years, we need a sample size of 143 per group to detect a difference in the primary endpoint. 286 patients in total need to be allocated to the two treatment arms. However, due to the nature of the disease, 30% of all patients will be dead before year two. Due to the in- and exclusion criteria we presume that only 20% of the study population will be dead before stratification. Thus 358 patients in total need to be included in the protocol. Data will be analysed according to the treatment the patients actually received using the Kaplan-Meier analysis. Patients will be analysed per protocol. New data have shown significant reduction in PBD after 4 years treatment with zoledronic acid compared to 2 years of treatment in 170 randomised patients^33^.

Other endpoints:

Serum samples will be collected and stored from all patients. From the calculations above, we expect 32 cases of PBD during year two to four. Bone markers will be measured on these patients from the time of PBD and 6 month prior to the bone event. An equal number of controls will be selected as the number of cases PBD actually observed and bone markers will be measured likewise in these patients. The controls will be selected according the stratification criteria’s (previous autologous stem cell treatment, presence or absence of bone disease at diagnosis, and previous treatment with bortezomib). If multiple options are present the same gender will be chosen, and after that the closest age related. Data will be analyzed as a case control study

Sites may participate in the study even if they only evaluate bone disease using either conventional radiography or low-dose CT. Since this part of the study is mainly descriptive, no statistical cut-off values have been defined. We expect however, that at least half of the patients will be evaluated using both modalities. All patients will have bone imaging conducted at the time points specified in appendix 1. Each patient will function as his or her own control since we will look for discrepancies between the different modalities, and the development in these discrepancies over time. Data will be analysed using the student t-test.

Amendment: The amendment will have no impact on the statistical consideration since all patients will be randomised. Only the part of the study comparing low-dose CT to conventional radiography will be affected. This part of the study however is descriptive and each patient function as his or her own control no statistical changes need to be made.

**1.14 Ethical Considerations**

Multiple myeloma remains an incurable malignant disorder; however the average expected lifetime is increasing due to improved treatment. Treatment monthly with zoledronic acid is known to reduce the risk pathological fractures, progressive osteolysis, and to reduce pain. No knowledge exists on what should be done after two years of treatment. Prolonged treatment carries the risk of BON, whereas discontinuation of treatment probably increases the risk of PBD. The primary purpose of the study is to investigate if prolonged treatment with zoledronic acid provides adequate protection against PBD to justify an increased risk of BON. To investigate this, patients will be randomized 1:1 to receive either 2 or 4 years of treatment. To protect participating patients from both under- overtreatment, routine oral cavity examinations and bone imaging are planned in the protocol. Bone imaging is balanced between early detection of PBD (and thus possible removal from the protocol) and minimal radiation exposure. It is estimated that a patients will receive an excess radiation dose of 33.2 – 36.6 mSv equal to the dose received from 1.5 conventional CT. This radiation exposure is not ignorable. The median age of a newly diagnosed patient however is approximately 70 years, they all suffer from an incurable cancer and have a severely reduced life expectancy. This reduces the long-term risk of secondary malignancies due to radiation exposure to approximately 0.0175%. Furthermore, the additional bone imaging will not only help us decide if the more sensitive modality low-dose CT in the future should replace conventional radiography, it will also help determining if serum samples partly can replace bone imaging, thus reducing future radiation exposure. Most importantly, it will in the actual patient help us detect progressive bone disease before this becomes symptomatic due to e.g. vertebral collapse or pathological fractures.

An additional part of the study is to investigate if serum bone markers can be used to tailor treatment with zoledronic acid in the individual patients in the future. To investigate this, extra serum will be collected and stored for later analysis. This extra serum will be collected when blood samples to monitor the myeloma disease are collected anyway, thus no additional appearances at the outpatient clinic for blood draws over the course of the study are anticipated.

REGULATORY ETHICS COMPLIANCE

The site investigator is responsible for ensuring that the clinical study is performed in accordance with the protocol, Good Clinical Practice (GCP), and applicable regulatory requirements at the given site.

Before the start of the study in a country, the national investigator will provide the national ethical committee with current and complete copies of the following documents:

• Final protocol and, if applicable, amendments

• Written patient information, informed consent form and quality of life questionnaires

• Any other documents that the ethical committee requests to fulfil its obligation

This study will be undertaken only after the ethical committee and the National Health and Medicines Authority have given full approval of the final protocol, any amendments, the informed consent form and applicable recruiting material, and the sponsor has received a copy of this approval. This approval letter must be dated and must clearly identify the documents being approved.

PATIENT INFORMATION AND INFORMED CONSENT

Each patient must give written consent after the nature of the study has been fully explained. The consent form must be approved by the ethical committee. The informed consent should be in accordance with principles that originated in the Declaration of Helsinki, current ICH and GCP guidelines, and applicable regulatory requirements.

Before entry into the study, the site-investigator or an authorized member of the investigational staff must explain to potential patients the aims, methods, reasonably anticipated benefits, and potential hazards of the study, and any discomfort it may entail. Patients will be informed that their participation is voluntary and that they may withdraw consent to participate at any time. They will be informed that choosing not to participate will not affect the treatment of his/her disease and will not prejudice future treatment. The patient will be given sufficient time (in most cases one day or longer) to read the informed consent form and the opportunity to ask questions. After having obtained the consent, a copy of the informed consent form must be given to the patient.

Based on a total assessment, it is the opinion of the steering committee that carrying out the study is ethically warranted.

Amendment: Patient included according to the protocol will be exposed to less radiation, thus the amendment should pose no ethical problems.

**1.15 Privacy of Personal Data and Data Handling**

Each patient will be assigned a personal identification number on inclusion. The data will be archived in accordance with GCP rules for 5 years in Denmark and Norway and for 10 years in Sweden following completion of the study. The collection and processing of personal data from patients enrolled in this study will be limited to

those data that are necessary to investigate the efficacy, safety, quality, and utility of the study drugs

used in this study.

The data will be recorded on case report forms (CRF). Patients will receive a protocol number for the CRF. A patient log which may pair the protocol number with the civic registration numbers will be kept under lock and key at the sponsor’s research facility. Since this is not a blinded study decoding of the patients will probably not be necessary. However if a situation arises where it is deemed in the best interest of the patient to break to code, this will have to be decided by the national investigator or the sponsor.

Bone markers will not be measured locally. Instead serum samples collected during the study will be stored locally and then later sent to The Haematological Research Unit in Odense, Denmark. Bone markers measured bi-annually will be analysed for all patients. As stated in section 1.13 32 cases and 32 controls will be selected and monthly bone markers will be analysed 6 months retrospectively in these patients. The serum samples are collected for this specific purpose and no in order to created a research biobank. For further details on the bone markers see appendix 2.

Radiological data will be mailed to The Haematological Research Unit in Odense, Denmark. They will be kept there for later review, as specified in appendix 3.

**1.16 Access to Source Data**

Sponsor, investigators, auditors, study nurses at participating departments, the national medicines agencies, the national data protection agencies, and the national committees on research ethics may be given access to the source data.

**1.17 Publication Policy**

Both positive and negative results will be publish in the best available international medical journal in order to obtain the widest possible impact. Authorship will be decided according to the ICMJE 2013 guidelines.

**1.18 Data Quality Assurance**

The project will be monitored by the national GCP unit in Denmark and by FoU-centrum, Kliniskt prövningsstöd, Skånes Universitetssjukhus – Lund, in Sweden and Norway

**1.19 Funding**

Treatment with zoledronic acid in multiple myeloma has already received EMEA approval and is an established treatment in all the participating countries. There has been no commercial interest from the pharmaceutical companies to participate in this study. A possible outcome of the study could be that the patients should receive less treatment with zoledronic acid. External funds will be applied for to cover the expenses in the study.

**1.20 Timescale**

Initiation is expected to occur end 2014, and due to new data inclusion will end 01.12.2020 with a follow up two years the expected termination date is 01.12.2022.

An interim analysis will be conducted at the end in inclusion. The steering committee will then decide if the positive effect of a decrease in the number of PBD outweighs a negative effect of an increased risk of BON, or if the study should be terminated.

**1.21 Insurance**

The patients are insured according to national standards. It is the responsibility of the national investigators to make sure the formalities are in place before patients are included in the respective country.

**1.22 References**

1. Kyle, R.A., et al., *Review of 1027 patients with newly diagnosed multiple myeloma.* Mayo Clin Proc, 2003. **78**(1): p. 21-33.

2. Terpos, E., et al., *The use of bisphosphonates in multiple myeloma: recommendations of an expert panel on behalf of the European Myeloma Network.* Ann Oncol, 2009. **20**(8): p. 1303-17.

3. Wisloff, F. and M. Hjorth, *Health-related quality of life assessed before and during chemotherapy predicts for survival in multiple myeloma. Nordic Myeloma Study Group.* Br J Haematol, 1997. **97**(1): p. 29-37.

4. Berenson, J.R., et al., *Efficacy of pamidronate in reducing skeletal events in patients with advanced multiple myeloma. Myeloma Aredia Study Group.* N Engl J Med, 1996. **334**(8): p. 488-93.

5. Rosen, L.S., et al., *Long-term efficacy and safety of zoledronic acid compared with pamidronate disodium in the treatment of skeletal complications in patients with advanced multiple myeloma or breast carcinoma: a randomized, double-blind, multicenter, comparative trial.* Cancer, 2003. **98**(8): p. 1735-44.

6. Morgan, G.J., et al., *First-line treatment with zoledronic acid as compared with clodronic acid in multiple myeloma (MRC Myeloma IX): a randomised controlled trial.* Lancet, 2010. **376**(9757): p. 1989-99.

7. Berenson, J.R., et al., *Long-term pamidronate treatment of advanced multiple myeloma patients reduces skeletal events. Myeloma Aredia Study Group.* J Clin Oncol, 1998. **16**(2): p. 593-602.

8. Morgan, G.J., et al., *Effects of zoledronic acid versus clodronic acid on skeletal morbidity in patients with newly diagnosed multiple myeloma (MRC Myeloma IX): secondary outcomes from a randomised controlled trial.* Lancet Oncol, 2011. **12**(8): p. 743-52.

9. Marx, R.E., *Pamidronate (Aredia) and zoledronate (Zometa) induced avascular necrosis of the jaws: a growing epidemic.* J Oral Maxillofac Surg, 2003. **61**(9): p. 1115-7.

10. Bamias, A., et al., *Osteonecrosis of the jaw in cancer after treatment with bisphosphonates: incidence and risk factors.* J Clin Oncol, 2005. **23**(34): p. 8580-7.

11. Zervas, K., et al., *Incidence, risk factors and management of osteonecrosis of the jaw in patients with multiple myeloma: a single-centre experience in 303 patients.* Br J Haematol, 2006. **134**(6): p. 620-3.

12. Montefusco, V., et al., *Antibiotic prophylaxis before dental procedures may reduce the incidence of osteonecrosis of the jaw in patients with multiple myeloma treated with bisphosphonates.* Leuk Lymphoma, 2008. **49**(11): p. 2156-62.

13. Dimopoulos, M.A., et al., *Reduction of osteonecrosis of the jaw (ONJ) after implementation of preventive measures in patients with multiple myeloma treated with zoledronic acid.* Ann Oncol, 2009. **20**(1): p. 117-20.

14. Ripamonti, C.I., et al., *Decreased occurrence of osteonecrosis of the jaw after implementation of dental preventive measures in solid tumour patients with bone metastases treated with bisphosphonates. The experience of the National Cancer Institute of Milan.* Ann Oncol, 2009. **20**(1): p. 137-45.

15. Kyle, R.A., et al., *American Society of Clinical Oncology 2007 clinical practice guideline update on the role of bisphosphonates in multiple myeloma.* J Clin Oncol, 2007. **25**(17): p. 2464-72.

16. Lund, T., et al., *Effect of withdrawal of zoledronic acid treatment on bone remodelling markers in multiple myeloma.* Br J Haematol, 2010. **151**(1): p. 92-3.

17. R. Garcia-Sanz, A.O., J. de la Rubia, L. Palomera, P. Ribas, MT. Hernandez, MJ. Moreno, J. Bargay, A. ramirez, Al. Teruel, M. Blanchard, M. Gironella, M. Granell, E. Abella, JF San Miguel. , *Analysis of zoledronic acid therapy for patients with multiple myeloma with asymptomatic biochemical relapse.* ASH abstract 2967, 2012.

18. Bataille, R., et al., *Recruitment of new osteoblasts and osteoclasts is the earliest critical event in the pathogenesis of human multiple myeloma.* J Clin Invest, 1991. **88**(1): p. 62-6.

19. Taube, T., et al., *Abnormal bone remodelling in patients with myelomatosis and normal biochemical indices of bone resorption.* Eur J Haematol, 1992. **49**(4): p. 192-8.

20. Terpos, E., et al., *The use of biochemical markers of bone remodeling in multiple myeloma: a report of the International Myeloma Working Group.* Leukemia, 2010. **24**(10): p. 1700-12.

21. Lund, T., et al., *Multiple myeloma: changes in serum C-terminal telopeptide of collagen type I and bone-specific alkaline phosphatase can be used in daily practice to detect imminent osteolysis.* Eur J Haematol, 2010. **84**(5): p. 412-20.

22. D'Sa, S., et al., *Guidelines for the use of imaging in the management of myeloma.* Br J Haematol, 2007. **137**(1): p. 49-63.

23. Kyle, R.A. and S.V. Rajkumar, *Criteria for diagnosis, staging, risk stratification and response assessment of multiple myeloma.* Leukemia, 2009. **23**(1): p. 3-9.

24. Dimopoulos, M.A., et al., *Risk of disease progression in asymptomatic multiple myeloma.* Am J Med, 1993. **94**(1): p. 57-61.

25. Mahnken, A.H., et al., *Multidetector CT of the spine in multiple myeloma: comparison with MR imaging and radiography.* AJR Am J Roentgenol, 2002. **178**(6): p. 1429-36.

26. Horger, M., et al., *Whole-body low-dose multidetector row-CT in the diagnosis of multiple myeloma: an alternative to conventional radiography.* Eur J Radiol, 2005. **54**(2): p. 289-97.

27. Gleeson, T.G., et al., *Accuracy of whole-body low-dose multidetector CT (WBLDCT) versus skeletal survey in the detection of myelomatous lesions, and correlation of disease distribution with whole-body MRI (WBMRI).* Skeletal Radiol, 2009. **38**(3): p. 225-36.

28. Princewill, K., et al., *Multiple myeloma lesion detection with whole body CT versus radiographic skeletal survey.* Cancer Invest, 2013. **31**(3): p. 206-11.

29. Advisory Task Force on Bisphosphonate-Related Ostenonecrosis of the Jaws, A.A.o.O. and S. Maxillofacial, *American Association of Oral and Maxillofacial Surgeons position paper on bisphosphonate-related osteonecrosis of the jaws.* J Oral Maxillofac Surg, 2007. **65**(3): p. 369-76.

30. Ruggiero, S.L., et al., *American Association of Oral and Maxillofacial Surgeons position paper on bisphosphonate-related osteonecrosis of the jaw - 2009 update.* Aust Endod J, 2009. **35**(3): p. 119-30.

31. Morgan, G.J., et al., *Effects of induction and maintenance plus long-term bisphosphonates on bone disease in patients with multiple myeloma: the Medical Research Council Myeloma IX Trial.* Blood, 2012. **119**(23): p. 5374-83.

32. Garcia-Sanz, R., *Analysis of Zoledronic Acid Therapy for Patients with Multiple Myeloma with Asymptomatical Biochemical Relapse* 2012.

33. Avilés et al. *Prolonged Use of Zoledronic Acid (4 Years)Did Not Improve Outcome in Multiple Myeloma Patients.* Clin Lymphoma Myeloma Leuk. 2017 Apr;17(4):207-210
